# Design and Implementation of a Flexible Photovoltaic Emulator Using a GaN-Based Synchronous Buck Converter

**DOI:** 10.3390/mi12121587

**Published:** 2021-12-20

**Authors:** Chao-Tsung Ma, Zhen-Yu Tsai, Hung-Hsien Ku, Chin-Lung Hsieh

**Affiliations:** 1 Applied Power Electronics Systems Research Group, Department EE, CEECS, National United University, Miaoli City 36063, Taiwan; M0721013@o365.nuu.edu.tw; 2 Chemistry Division, Institute of Nuclear Energy Research (INER), AEC, Executive Yuan, Taoyuan 325207, Taiwan; HHKu@iner.gov.tw (H.-H.K.); clhsieh@iner.gov.tw (C.-L.H.)

**Keywords:** wide bandgap semiconductor, gallium nitride, photovoltaic module, digital signal processor, synchronous buck converter

## Abstract

In order to efficiently facilitate various research works related to power converter design and testing for solar photovoltaic (PV) generation systems, it is a great merit to use advanced power-converter-based and digitally controlled PV emulators in place of actual PV modules to reduce the space, cost, and time to obtain the required scenarios of solar irradiances for various functional tests. This paper presents a flexible PV emulator based on gallium nitride (GaN), a wide-bandgap (WBG) semiconductor, and a based synchronous buck converter and controlled with a digital signal processor (DSP). With the help of GaN-based switching devices, the proposed emulator can accurately mimic the dynamic voltage-current characteristics of any PV module under normal irradiance and partial shading conditions. With the proposed PV emulator, it is possible to closely emulate any PV module characteristic both theoretically, based on manufacturer’s datasheets, and experimentally, based on measured data from practical PV modules. A curve fitting algorithm is used to handle the real-time generation of control signals for the digital controller. Both simulation with computer software and implementation on 1 kW GaN-based experimental hardware using Texas Instruments DSP as the controller have been carried out. Results show that the proposed emulator achieves efficiency as high as 99.05% and exhibits multifaceted application features in tracking various PV voltage and current parameters, demonstrating the feasibility and excellent performance of the proposed PV emulator.

## 1. Introduction

It has been predicted that the world’s energy consumption will increase by roughly 28% between 2015 and 2040, with residential and commercial buildings’ electricity usage being the major contributor to the growth [1]. Conventional electricity generation technologies tend to produce carbon dioxide emission, which is believed to be associated with global warming and some other environmental issues. This has led to an urgent need to increase the penetration of renewable energy (RE)-based distributed power generation (DG). In the past decade, the penetration of typical RE-based DG technologies, such as wind power generation and solar photovoltaic (PV) generation, has grown drastically worldwide [2]. Because of the inherently unpredictable and nonlinear characteristics of these RE-based DG technologies, improvement in energy harvesting and conversion technologies is much desired. Among currently used RE sources, solar PV offers many advantages: it is inexhaustible and pollution-free, and it exhibits the highest energy density, with an average of 170 W/m^2^. In addition, solar PV generation systems require relatively low maintenance costs. Increasing the usage of distributed PV systems to a certain level can substantially reduce transmission losses, thus increasing energy utilization. Based on the data reported in [3], until 2017, the total share of global RE-based generation was only roughly 4%, but it has been expected that the solar PV generation level will catch up in the share of future RE-based generation.

The PV cells in a PV module produce DC electricity out of sunlight. As a result, a proper power converter interface is required for a grid-connected or standalone solar PV generation system when supplying electricity to AC loads. When power converters are tested for practical PV generation applications, environmental conditions such as solar irradiance and ambience temperature have to be considered. Testing the converters under actual and various environmental conditions is a valid yet time-consuming method, and the associated cost may be very high. Moreover, it is also challenging to obtain the current and voltage (I-V) characteristics of a PV module in a laboratory with limited space. A better approach is to design PV emulators using power converters, which require much less space and are easily programmable. In the literature, many PV emulation systems have been reported. In [4], a buck converter-based PV emulator was controlled using a fast-convergence resistance-feedback strategy that required a current-resistance model of a PV module. A dynamic field programmable analog array (FPAA)-based PV emulator was presented in [5] with the following advantages: (1) it did not require numerical interpolation or large amount of data in memory; (2) it was easily characterized, compared with a field programmable gate array (FPGA)- or digital signal processor (DSP)-based implementations. In [6], a portable, inexpensive and light-weight plug-and-play PV emulator was developed using a DC/DC converter. Great promise for PV module emulation with a minimum amount of output error was shown. In [7], S. M. Azharuddin et al. proposed a near-accurate PV emulator using a simple power converter and a dSPACE controller for real-time control. The proposed device enabled fast converter response with a high bandwidth current regulator. A low-cost, portable, embedded design for implementing a real-time, high-accuracy PV emulator using new processors was proposed in [8].

For industrial applications, a PV emulator with a higher power rating is required. In [9], a dynamic 10-kVA PV emulator based on high-power inverters and an active LCL filter was developed and implemented. The maximum power point tracking (MPPT) algorithms of a real PV module were tested using the proposed hardware. The minimized harmonic current content and fast adjustment for power factor (PF) were achieved with the selected power stage. A. Koran et al. [10] proposed a high-efficiency PV source simulator with the advantages of using analog and digital-based controllers. A three-phase AC-DC dual boost rectifier cascaded with a three-phase DC-DC interleaved buck converter was used in this system. A low-cost PV module emulation system developed using LabVIEW software was proposed in [11]. Several analytical models of PV cells and the estimation of power and current curves for a given irradiance and temperature were studied. D. Pelin et al. [12] described a PV emulator using DC programmable sources and conducted a 6-day emulation. In [13], a real-time emulator for control and diagnosis purposes of solar PV generation was developed in a Matlab/Simulink environment. I-V characteristics of generator model were emulated with a programmable DC/AC power source. A PV emulator with quasi-sliding mode controller to realize PV characteristics under rapidly varying environmental conditions was proposed in [14]. In their design case, a user-friendly graphical user interface (GUI) was developed. In [15], a PV emulator using dSPACE controller with simple control was designed to achieve fast response. Current mode controller and a modified look-up table were combined for the control mechanism.

In most of the papers reviewed above, the theoretical models using V-I curves of the given PV modules and the conventional silicon (Si)-based switching devices were commonly used for designing converter-based PV emulators. In the literature, a conference paper [16] presented a hardware design case of the PV module emulator, in which GaN MOSFETs were used; however, the details of controller design issues were not discussed. Comprehensive analysis and comparison of performance between Si- and GaN-based DC-DC converters can be found in [17]. In this paper, a gallium nitride (GaN)-based and DSP-controlled DC-DC converter is presented to achieve a flexible and highly efficient PV emulator with the capability to closely simulate any V-I characteristic of a PV module using parameters either from manufacture’s datasheets or measured data, including normal operating conditions and shading conditions. Flexible emulation features have been implemented with the proposed digital control scheme and verified with a 1 kW hardware prototype. This paper is organized into four sections. In the next section, the PV model is firstly derived, from the cell level to the module level. Then, the modeling of the DC/DC converter and controllers, along with the implementation methodology, are explained. Section 3 presents results from the simulation and implementation of the proposed emulator with an electronic load to demonstrate the performance of the proposed PV emulator. The test of the system efficiencies of the proposed PV emulator using GaN-based synchronous buck converter operated at different switching frequencies is presented in Section 4. Finally, a summary of the paper is given in the last section.

## 2. PV Module Modeling and Design of Synchronous Buck Converter

### 2.1. PV Module Modeling

**Figure 1:**
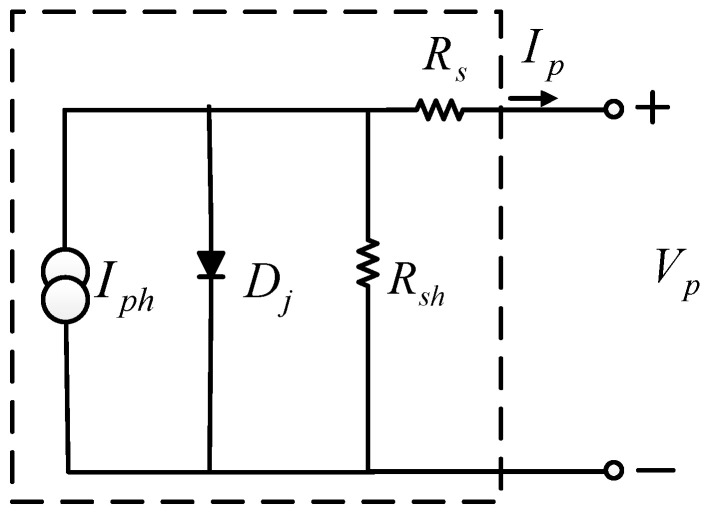
Equivalent circuit of a PV cell.

A PV module consists of a number of PV cells in series and parallel. Each PV cell is made of a P-N junction semiconductor diode. As the equivalent circuit shown in Figure 1, *I_ph_* represents equivalent current source (A); *D_j_* represents the P-N junction diode; *R_sh_* represents equivalent parallel resistance of the material (Ω); *R_s_* represents equivalent series resistance of the material (Ω), and *V_p_* and *I_p_* represent output voltage (V) and current (A), respectively.

From Figure 1, the relationship between output voltage and current can be derived as follows:

(1) $$I_{p}=I_{ph}-I_{rs}\cdot \left\{\exp\left[\frac{q(V_{p}+R_{s}I_{p})}{AkT}\right]-1\right\}-\frac{V_{p}+R_{s}I_{p}}{R_{sh}},$$

where *I_rs_* represents equivalent reverse saturated current of *D_j_* (A); *q* represents elementary charge (1.60 × 10^−19^ C); *A* represents ideal factor (ranging from 1 to 5); *k* represents Boltzmann constant (1.38 × 10^−23^ J/K), and *T* represents surface temperature (K).

**Figure 2:**
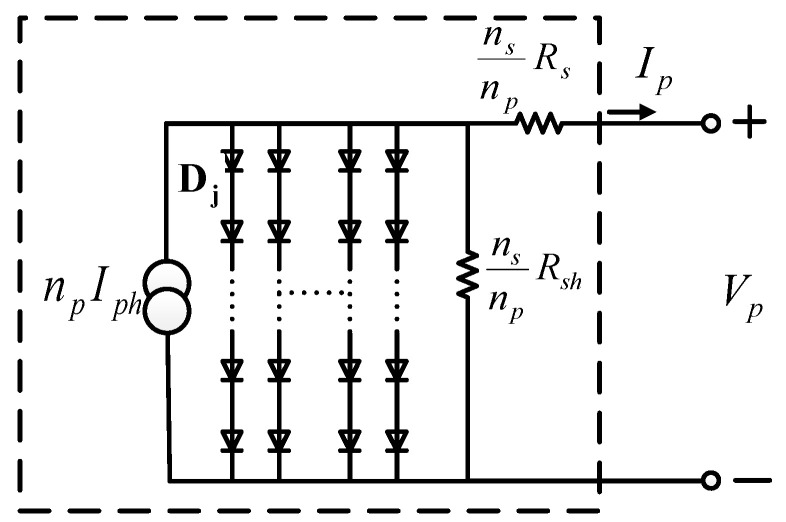
Equivalent circuit of a PV module.

Because the voltage, current, and power generated by a single PV cell are low, many PV cells are usually connected in series and parallel to form a PV module in order to boost the output, as the equivalent circuit shown in Figure 2, where *n_p_* represents the number of paralleled diode paths, and *n_s_* represents the number of series-connected diodes in each path.

Under normal conditions, *R_sh_* is large, and *R_s_* is small. Ignoring these two parameters, we can simplify (1) to obtain (2).

(2) $$I_{p}=I_{ph}-I_{rs}\cdot [\exp\left(\frac{qV_{p}}{AkT}\right)-1],$$

where

(3) $$I_{rs}=I_{rr}\cdot {\left(\frac{T}{T_{r}}\right)}^{3}\cdot \exp[\frac{qE_{g}}{kA}(\frac{1}{T_{r}}-\frac{1}{T})],$$

*I_rr_* represents reverse saturated current (A) at reference temperature (*T_r_*), and

(4) $$E_{g}=1.16-(7.02\times 10^{-4})\cdot \frac{T^{2}}{T-1108},$$

Also,

(5) $$I_{ph}=[I_{scr}+\frac{\alpha }{1000}(T-T_{r})]\cdot S,$$

where *I_scr_* represents short-circuit current (A) at reference temperature and irradiance; *α* represents short-circuit temperature coefficient of a PV cell (mA/°C), and *S* represents solar irradiance (kW/m^2^). The output power of a PV cell can then be expressed as follows:

(6) $$P_{p}=V_{p}I_{p}=I_{ph}V_{p}-I_{rs}V_{p}\cdot [\exp(\frac{qV_{p}}{AkT})-1].$$

In Figure 2, *R_sh_* and *R_s_* can be ignored so that the relationship between output voltage and current can be expressed as follows:

(7) $$I_{p}=n_{p}I_{ph}-n_{p}I_{rs}\cdot [\exp(\frac{qV_{p}}{AkTn_{s}})-1].$$

As a result, the output power of the PV module can then be expressed as follows:

(8) $$P_{p}=V_{p}I_{p}=n_{p}I_{ph}V_{p}-n_{s}I_{rs}V_{p}\cdot [\exp(\frac{qV_{p}}{AkTn_{s}})-1]$$

**Figure 3:**
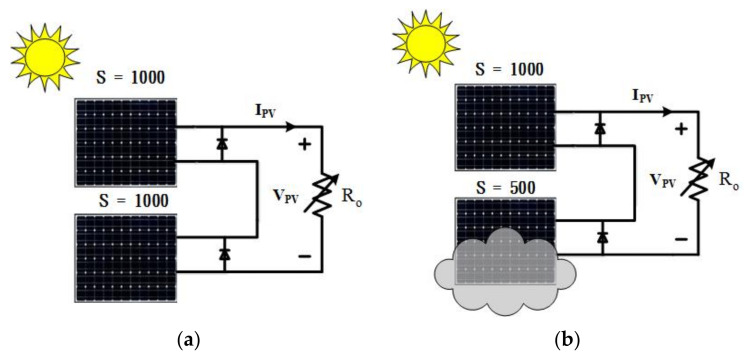
Schematic configuration of two PV modules connected in series, (**a**): *S* = 1000/1000, (**b**): S = 1000/500.

In this paper, two PV modules are evaluated for the design of the PV emulator. The specifications of a single PV module are as follows: rated power *P_mp_* = 300 W, number of PV cells = 60, rated current *I_mp_* = 7.87 A, rated voltage *V_mp_* = 38.4 V, short-circuit current *I_sc_* = 8.7 A, open-circuit voltage *V_oc_* = 48 V, and idea factor *A* = 1.5. The ambience temperature is set at 25 °C. To evaluate the V-I characteristics of multiple PV modules connected in series under normal and partial shading scenarios, two PV modules are used, as shown in Figure 3, where the output terminals of each module are shunted with a bypass diode.

**Figure 4:**
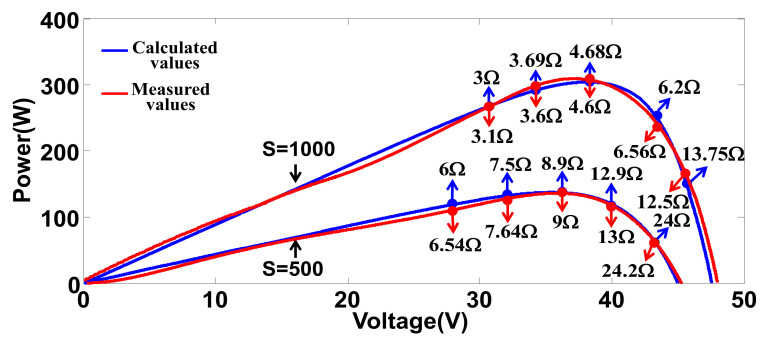
Comparison of calculated and measured P-V characteristics of a single PV module under 0–150 Ω load conditions and with the same irradiance conditions (*S* = 1000 and 500).

**Figure 5:**
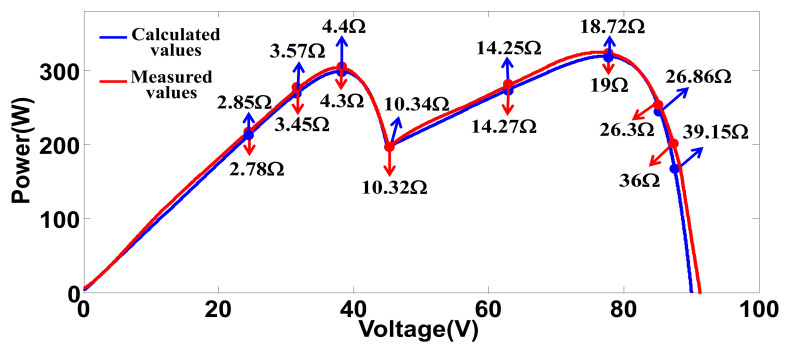
Comparison of calculated and measured P-V characteristics of two PV modules connected in series under 0–150 Ω load conditions and irradiance condition of *S* = 1000 and 500 for the two modules, respectively.

By applying the specifications to the derived equations and designating load and irradiance conditions, theoretical P-V characteristics of the PV modules can be calculated. Figure 4 shows theoretical and measured P-V characteristics of a single PV module under 0–150 Ω load conditions and two irradiance conditions (1000 and 500 W/m^2^, respectively). Next, Figure 5 shows theoretical and measured P-V characteristics of two PV modules connected in series under the same load conditions and one module receives 500 W/m^2^ irradiance, and the other receives 1000 W/m^2^ irradiance.

As can be seen in Figure 4 and Figure 5, the measured value and calculated values are not perfectly meet. This is dure to the inevitable error in curve fitting a limited number of measured points. Some advanced algorithms can be used to improve the quality of fitting but the calculating burden of digital controller will be increased.

### 2.2. Design of Synchronous Buck Converter

#### 2.2.1. Operating Principle and Mathematical Model of Synchronous Buck Converter

**Figure 6:**
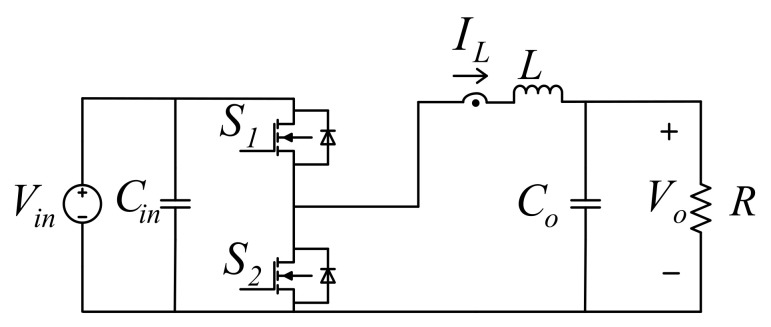
Configuration of synchronous buck converter.

Figure 6 shows the circuit configuration of the synchronous buck converter used in this paper, including two power switches *S*_1_ and *S*_2_, an inductor *L*, an input capacitor *C_in_*, and an output capacitor *C_o_*. The operating principle of this converter is as follows: *S*_1_ acts as the main switch; when it is on, *V_in_* charges L, and when S_1_ is off, the energy stored in *L* is transmitted through *S_2_* to *C_o_*.

State average method can be used to derive the mathematical model of the synchronous buck converter. When *S*_1_ is on, and *S*_2_ is off, the voltage across *L* can be expressed as follows:

(9) $$V_{L}=L\frac{di_{L}}{dt}=V_{in}-V_{o}.$$

When *S*_1_ is off, and *S*_2_ is on, the voltage across *L* can be expressed as follows:

(10) $$V_{L}=L\frac{di_{L}}{dt}=-V_{o}.$$

As a result, average inductor voltage can be expressed as follows:

(11) $$\begin{array}{ll} \bar{V_{L}(t)} & =L\frac{\bar{di_{L}}}{dt}=\frac{1}{T_{s}}\int_{0}^{T_{s}}V_{L}(\tau )d\tau \\ & =V_{L}(\tau 1)\frac{t_{1}}{T_{s}}+V_{L}(\tau 2)\frac{T_{s}-t_{1}}{T_{s}}\\ & =V_{L}(\tau 1)d+V_{L}(\tau 2)d^{\prime \prime } \end{array}$$

Substituting (9) and (10) into (11) gives the following:

(12) $$\begin{array}{ll} \bar{V_{L}(t)} & =(V_{in}-V_{R})d+(-V_{R})d^{\prime \prime }\\ & =dV_{in}-V_{R} \end{array}$$

Next, we consider the following disturbance terms:

(13) $$i_{L}=I_{L}+{\hat{i}}_{L}(t),\left|I_{L}\right|>>\left|{\hat{i}}_{L}(t)\right|;$$

(14) $$d=D+\hat{d}(t),\left|D\right|>>\left|\hat{d}(t)\right|;$$

(15) $$v_{in}=V_{in}+{\hat{v}}_{in}(t),\left|V_{in}\right|>>\left|{\hat{v}}_{in}(t)\right|;$$

(16) $$v_{o}=V_{o}+{\hat{v}}_{o}(t),\left|V_{o}\right|>>\left|{\hat{v}}_{o}(t)\right|.$$

As a result, (12) can be expressed as follows:

(17) $$\begin{array}{ll} L\frac{d(I_{L}+{\hat{i}}_{L}(t))}{dt} & =(D+\hat{d}(t))\cdot (V_{in}+{\hat{v}}_{in}(t))-(V_{o}+{\hat{v}}_{o}(t))\\ & =[DV_{in}-V_{o}]+[D{\hat{v}}_{in}(t)+V_{in}\hat{d}(t)-{\hat{v}}_{o}(t)]+[\hat{d}(t){\hat{v}}_{in}(t)] \end{array}$$

where $\hat{d}(t){\hat{v}}_{in}(t)$ is very small and thus can be neglected, and

(18) $$L\frac{d{\hat{i}}_{L}(t)}{dt}=D{\hat{v}}_{in}(t)+V_{in}\hat{d}(t)-{\hat{v}}_{o}(t).$$

Performing Laplace transform on (18) gives the following:

(19) $$sL{\hat{i}}_{L}(s)=D{\hat{v}}_{in}(s)+V_{in}\hat{d}(s)-{\hat{v}}_{o}(s).$$

**Figure 7:**
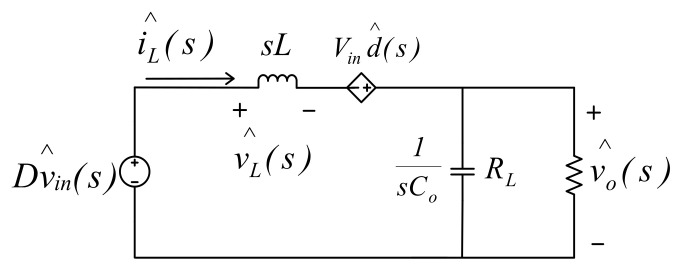
Mathematical model of synchronous buck converter.

As a result, we get the mathematical model shown in Figure 7.

#### 2.2.2. Design of Controllers for the Proposed PV Emulator

**Figure 8:**
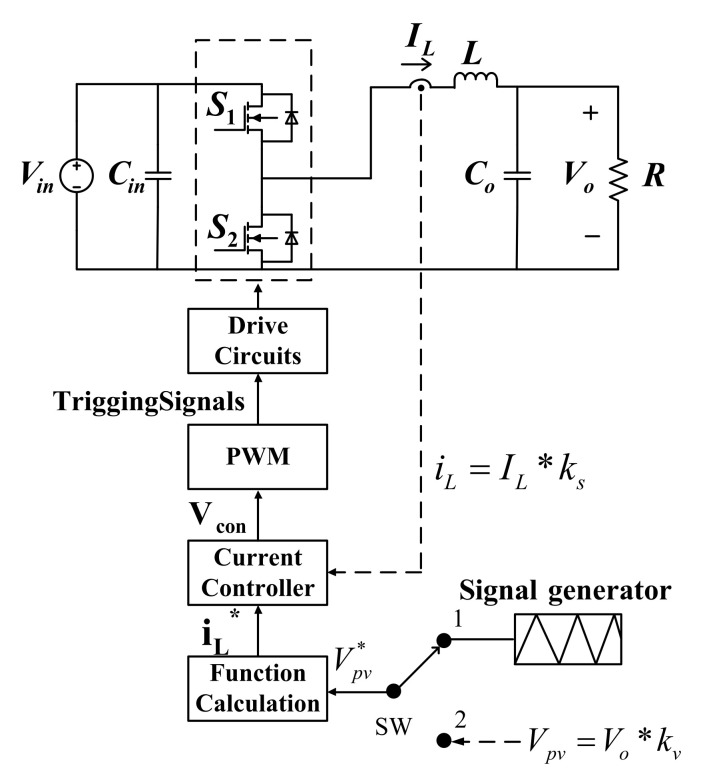
Block diagram of current mode control of synchronous buck converter.

The synchronous buck converter proposed in this paper adopts single-loop inductor current control. A signal generator is first used to generate a triangular wave, which is used to calculate inductor current command, and the current command is then compared with current feedback to yield the error signal, which is used to generate PWM control voltage. Finally, trigger signal is generated to adjust the duty cycle of the switches. Figure 8 shows the converter architecture, where *k_s_* and *k_v_* represent current and voltage sensing scales, respectively; *I_L_* and i_L_^*^ represent current feedback and command, respectively, and *v_con_* represents PWM voltage.

Letting ${\hat{v}}_{in}(s)=0$, we get the following:

(20) $$sL{\hat{i}}_{L}(s)-V_{in}\hat{d}(s)+{\hat{i}}_{L}(s)(\frac{1}{sC_{O}}//R_{L})=0.$$

As a result,

(21) $$\frac{{\hat{i}}_{L}(s)}{\hat{d}(s)}=\frac{V_{in}}{\left(sL+\frac{R_{L}}{1+sC_{O}R_{L}}\right)}=\frac{V_{in}(1+sC_{O}R_{L})}{sL(1+sC_{O}R_{L})+R_{L}}=\frac{V_{in}}{L}\cdot \frac{s+\frac{1}{C_{O}R_{L}}}{s^{2}+\frac{1}{C_{O}R_{L}}s+\frac{1}{C_{O}L}}.$$

**Figure 9:**
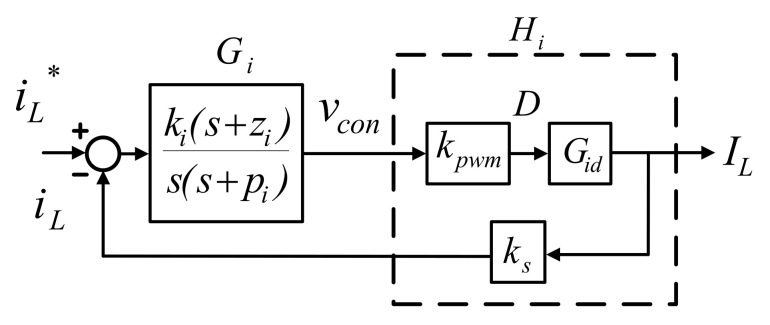
Block diagram of inductor current control loop.

Consequently, the block diagram of inductor current control loop can be graphed as shown in Figure 9, where type II proportional-integral (PI) controller and K factor are used to perform further quantification design.

According to Figure 9, we can obtain plant transfer function of the current control loop:

(22) $$\begin{array}{ll} H_{i}(s) & =k_{PWM}\cdot G_{id}\cdot k_{s}=\frac{1}{v_{t}}\times \frac{{\hat{i}}_{b}(s)}{\hat{d}(s)}\times k_{s}\\ & =\frac{k_{s}V_{in}}{v_{t}L}\times \frac{s+\frac{1}{R_{L}C_{O}}}{s^{2}+\frac{1}{R_{L}C_{O}}s+\frac{1}{LC_{O}}} \end{array}$$

where *R_L_* represents load resistance. Substituting the related design specifications into (22) gives the following:

(23) $$H_{i}(s)=\frac{6400s+5.77\times 10^{6}}{s^{2}+901.6s+2\times 10^{7}}.$$

Crossover frequency is chosen at 1/8 times the switching frequency:

(24) $$\omega_{ci}=f_{S}\times \frac{1}{8}\times 2\pi =50K\times \frac{1}{8}\times 2\pi =39250\left(\mathrm{rad}/s\right).$$

Consequently, we can calculate the gain and phase angle of the plant and the phase boost required at the crossover frequency:

(25) $$H_{i}(\omega_{i})=Gain_{Hi}∠Angle_{Hi}=0.16551∠-90^{\circ }.$$

(26) $$\begin{array}{ll} PhaseBoost & =PhaseMargin-Angle_{Hi}-90^{\circ }\\ & =80^{\circ }-(-90)-90^{\circ }=80^{\circ } \end{array}$$

K factor can then be calculated as follows:

(27) $$K_{factor}=tan(\frac{PhaseBoost}{2}+45^{\circ })=tan(\frac{80^{\circ }}{2}+45^{\circ })=11.43.$$

As a result, we get controller zero and pole:

(28) $$z=\frac{\omega_{ci}}{K_{factor}}=\frac{39250}{11.43}≅3433.95\left(\mathrm{rad}/S\right).$$

(29) $$p=\omega_{ci}\times K_{factor}=39250\times 11.43≅448627.5\left(\mathrm{rad}/S\right).$$

Required gain compensation at the crossover frequency is as follows:

(30) $$k_{i}=\frac{p}{Gain_{Hi}}=\frac{448627.5}{0.16551}≅2710576.4.$$

Finally we obtain the controller transform function according to (28)–(30):

(31) $$G_{i}(s)=\frac{k_{i}(s+z)}{s(s+p)}=\frac{2710576.4(s+3433.95)}{s(s+448627.5)}.$$

**Figure 10:**
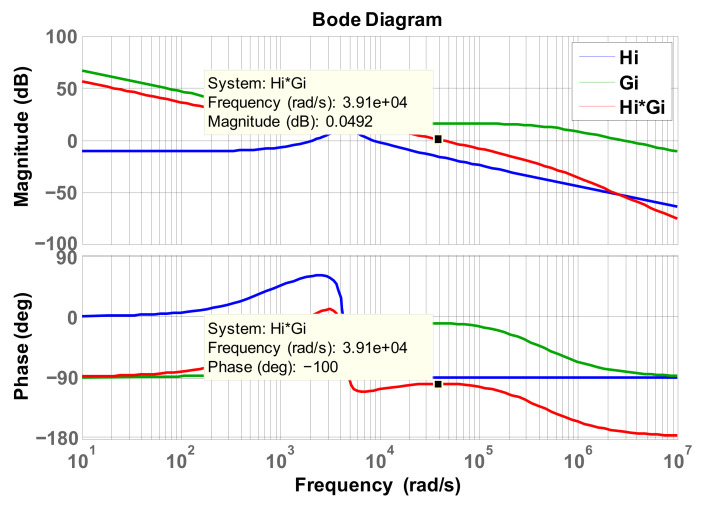
Bode plot of inductor current control loop.

Figure 10 shows Bode plot of the current control loop. The phase margin is 80 degrees.

## 3. Simulation and Implementation

To validate the performance of the designed PV emulator, both simulation studies with computer software and hardware implementation are performed.

**Figure 11:**
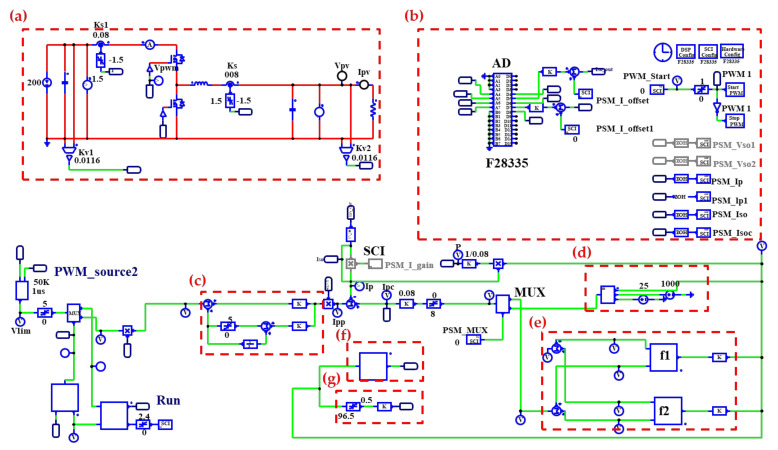
Complete simulation model of the proposed PV emulator and the arrangement of control signals. (**a**): buck-boost converter, (**b**): analog to digital module, (**c**): current controller, (**d**) and (**e**): PV model blocks, (**f**) and (**g**): voltage command blocks.

Figure 11 shows the complete software simulation model of the proposed PV emulator and the arrangement of control signals. In this paper, two control methods, the direct referencing method (case 1 and case 2) and output voltage feedback method (case 3), are used to implement the control of the proposed PV emulator with a digital control scheme. In Figure 11, block (a) is the power circuit of the proposed synchronous buck converter. Block (b) is an analog to digital (AD) module in TI’s DSP. Block (c) is the designed type-II current controller for tracking the current commands sent from PV model blocks (d) or (e). Block (d) is a program block interpreting the theoretical voltage-current relationship of emulated PV module as given in equation (7). Block (e) has two program blocks respectively describing the shedding conditions of two PV modules having different irradiances, e.g., S = 1000 and S = 500. In this study, a simple curve fitting method is used to fit the measured points (V & I pairs) of a given PV module and irradiance condition. In this study case, the number of measured voltage and current data pair is 10. It can be predicted that the measured and theoretically calculated values cannot be perfectly meet. This is due to the inevitable error in curve fitting a limited number of measured points. Some advanced algorithms can be used to improve the quality of fitting but the calculating burden of digital controller will be increased. In block (f), a voltage ramp generator is programmed to send out the emulated terminal voltages of the PV module to the PV model blocks, (e) and (d), for calculating the corresponding currents. As can be seen in Figure 11, the current commands of the PV emulator can be any combination of the outputs from (d) and (e), depending on the desired emulation scenario. It should be noted that, normally, a properly designed user interface is required for the practical application of the PV emulator; however, it is out of the investigation scope of the present paper.

**Figure 12:**
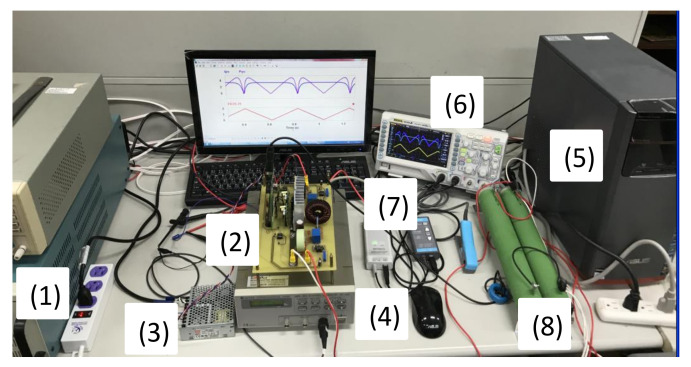
Complete hardware implementation of the proposed PV module emulator.

A photo of the complete hardware implementation of the DC-DC synchronous buck converter-based PV module emulator is shown in Figure 12, including (1) DC power supply, (2) DSP-controlled GaN based synchronous buck converter, (3) auxiliary power, (4) function generator, (5) personal computer, (6) digital oscilloscope, (7) voltage and current probes, and (8) resistive load. The synchronous buck converter is built using GaN power switches (TPH3207WS) with properly designed output inductor and capacitor values. Laboratory-made sensing circuits are used for voltage and current measurements. During operation, sensed current and voltage feedbacks are directly fed into the ADC ports of TI’s TMS320F28335 controller. Corresponding PWM pulses are then sent out via the PWM port. Gate pulses are isolated from the buck converter’s power circuitry with proper driving IC before being applied to the gate of the GaN switches. The major advantage of using DSP and a digital control scheme is online parameter tuning and better flexibility in implementing possible changes of conditions. To verify the effectiveness and performance of the proposed PV emulator, two sets of dynamic V-I characteristics and conditions, i.e., normal irradiance and partial shading conditions, are tested. It is found that the response time of the proposed controller is very short and that the absolute error between experimental results and calculated values is quite small. Typical results are presented in the following paragraphs for comparison.

**Figure 13:**
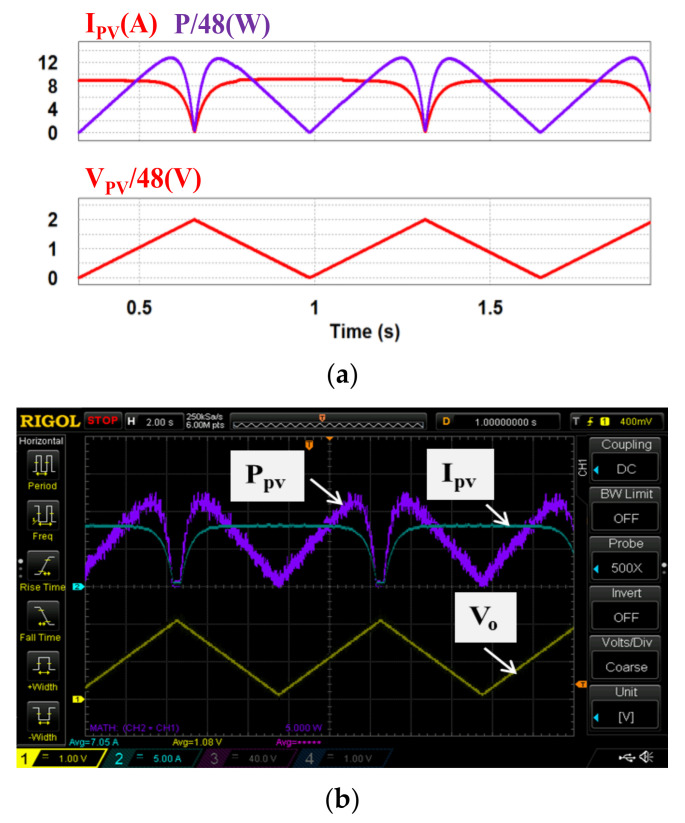
Experimental results of the proposed PV emulator under S = 1000 & 1000: (**a**) simulated power and current outputs and PV voltage; (**b**) implemented power and current outputs and PV voltage.

In case 1, theoretical characteristics of the two PV modules under normal irradiance (S = 1000 & 1000) are used. Figure 13a,b shows a set of simulation and measured results from the PV emulator (*I_PV_*: 5 A/div; *V_o_*: 40 V/div; *T*: 2 s/div; *V_PV_*: 48 V/div; *P_PV_*: 270 W/div).

**Figure 14:**
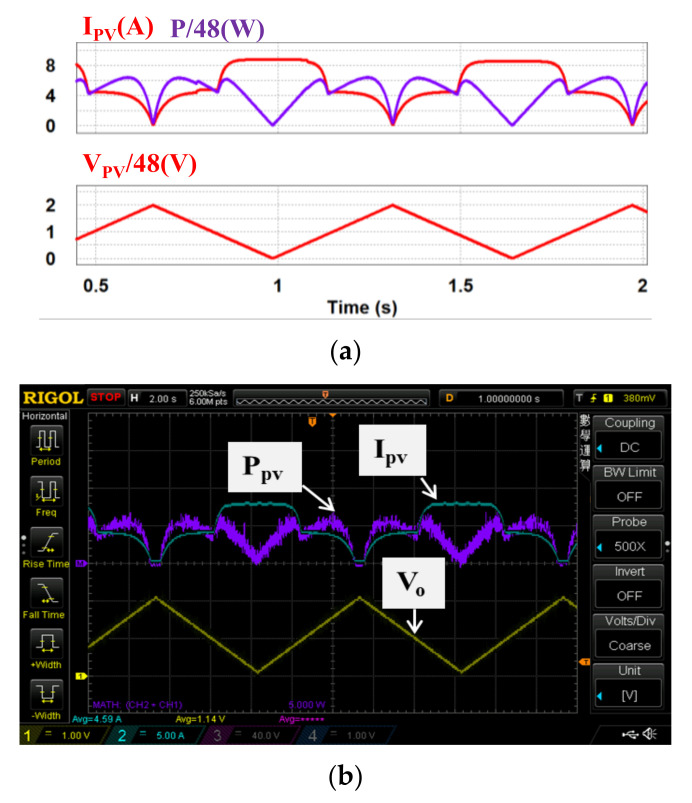
Output results of the proposed PV emulator under S = 500 & 1000 with theoretical PV values: (**a**) simulated power and current outputs and PV voltage; (**b**) implemented power and current outputs and PV voltage.

**Figure 15:**
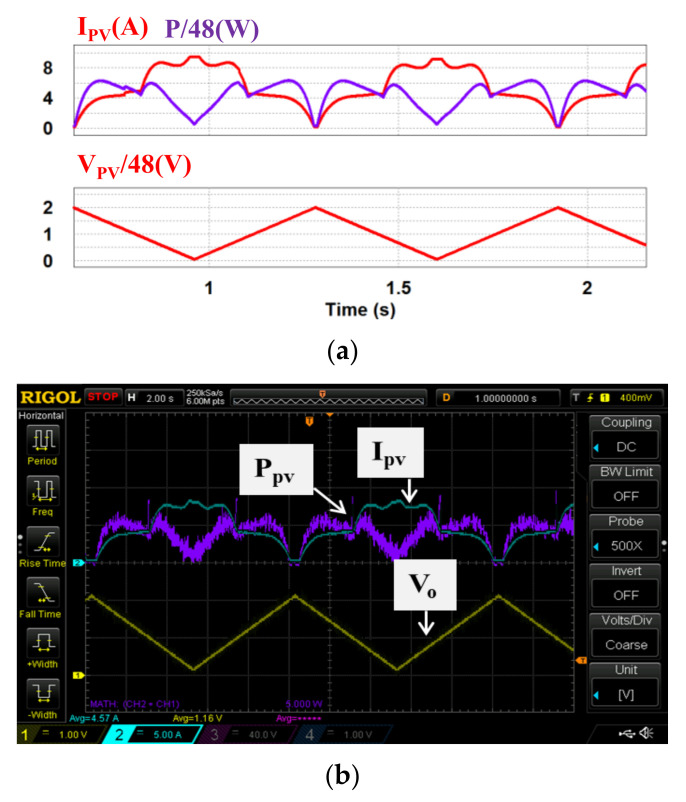
Output results of proposed PV emulator under *S* = 500 & 1000 with practical measured PV data: (**a**) simulated power and current outputs and PV voltage; (**b**) implemented power and current outputs and PV voltage.

In case 2, both theoretical and measured characteristics of the two PV modules under partial shading (S = 500 & 1000) are used. Figure 14a,b and Figure 15a,b respectively show the two sets of simulation and measured results from the proposed PV emulator (*I_PV_*: 5 A/div; *V_o_*: 40 V/div; *T*: 2 s/div; *V_PV_*: 48 V/div; *P_PV_*: 270 W/div).

**Figure 16:**
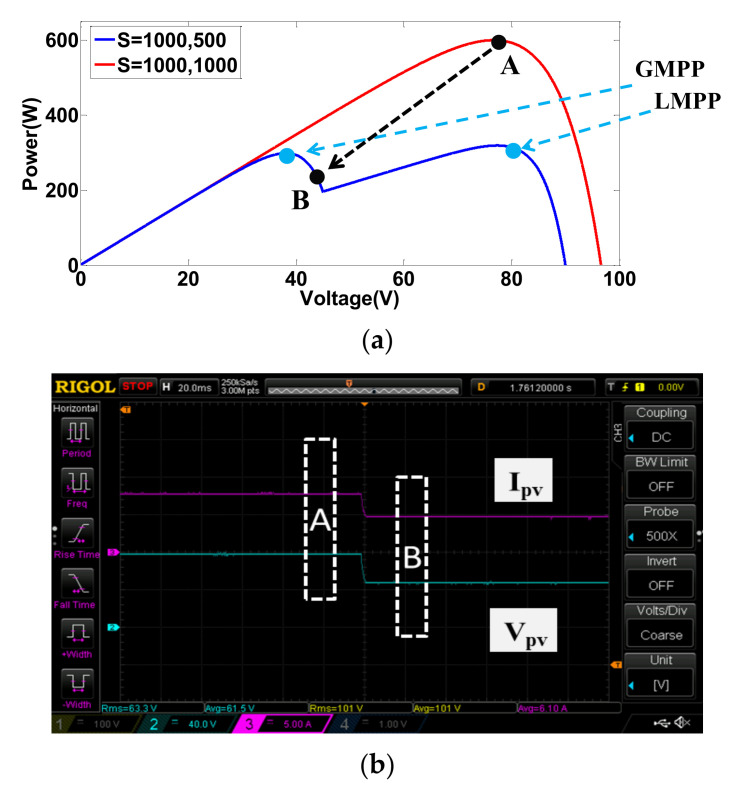
Results of transition from normal condition (A) to shading condition (B): (**a**) simulation; (**b**) implementation.

**Figure 17:**
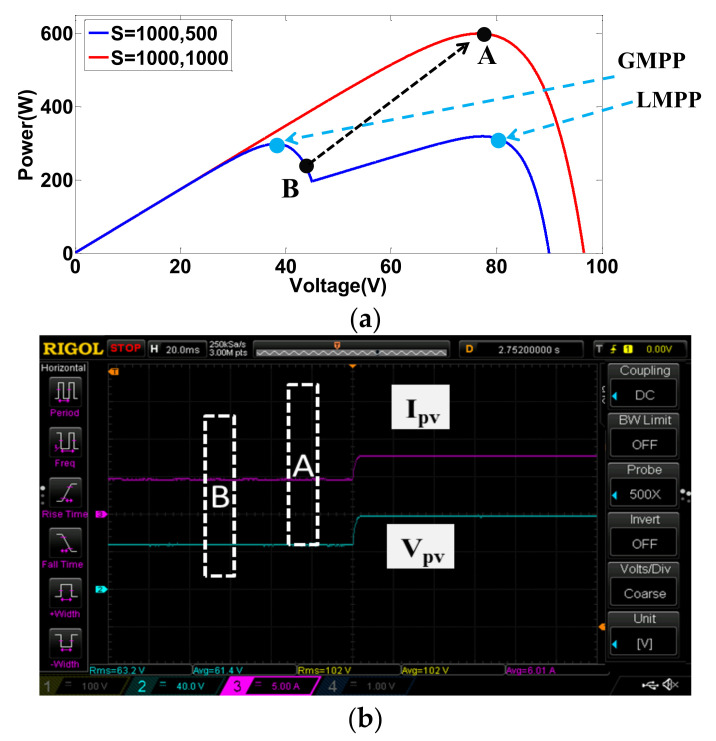
Results of transition from shading condition (B) to normal condition (A): (**a**) simulation; (**b**) implementation.

In case 3, the control transition between the conditions of cases 1 and 2 is performed. Figure 16a shows simulated transition from normal condition (A) to shading condition (B), and Figure 16b shows the corresponding measured waveforms. Figure 17 shows the results of control transition from shading condition (B) to normal condition (A) (*I_PV_*: 5 A/div; *V_pv_*: 40 V/div; *T*: 20 ms/div).

As can be seen in Figure 13, Figure 14, Figure 15, Figure 16 and Figure 17, performance in tracking the voltage-current parameters of a given PV module is high. It has been observed that the control error in all test cases is almost negligible. In control practice, the control error can be affected by a number of factors. Typical ones include the type of controller used, the precision of sensors used (the current sensors in this specific design case), the sampling speed, the level of EMI and the quality of the AD device, etc. In this design case, a type II controller, equivalent to a PI controller with a low-pass filter, is utilized to guarantee a theoretical zero-control-error. The accuracy of the Hall current sensor used in our design is about 0.2–1%. To eliminate the 0.2–1% sensing error, a homemade digital compensator is used at the AD configuring module of the TI’s DSP controller with a pre-tuning step.

## 4. The Analysis of System Efficiency

**Figure 18:**
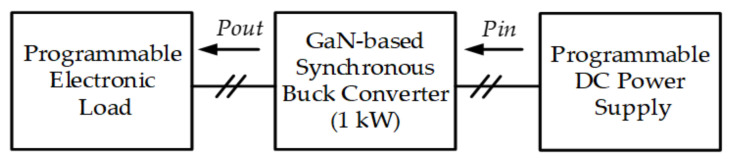
The system block diagram of the efficiency tests.

**Figure 19:**
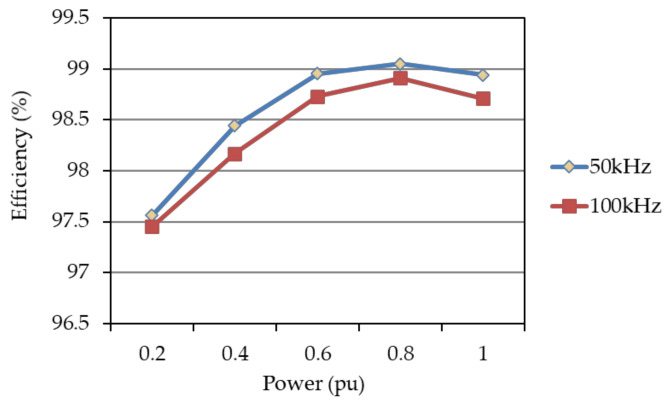
Efficiencies of the proposed GaN-based synchronous buck converter at different switching frequencies.

In this paper, the system efficiency of the proposed PV emulator using a GaN-based synchronous buck converter operated at different switching frequencies (50 kHz, 80 kHz) and different output power levels is practically tested. Figure 18 shows the system block diagram of the efficiency tests carried out in this study. In this test, the DC terminals of the proposed GaN-based synchronous buck converter are connected to a programmable DC power supply having the output voltage set to 200 V, and the DC output terminal voltage of the proposed GaN-based synchronous buck converter is regulated at a fixed 100 V by the proposed voltage controller. For testing the converter efficiency under different output power levels, a programmable electronic load is connected to the DC output terminal of the converter. By setting different *P_out_* and measuring the corresponding *P_in_* of the converter, the system efficiency at a specific power level and switching frequency can be readily calculated. In this paper, two switching frequencies, i.e., 50 and 80 kHz, are tested at five load levels. The calculated results are graphically shown in Figure 19. As can be seen in Figure 19, a maximum efficiency of 99.05% appears at about 80% of the converter’s rated capacity (1 kW), with a switching frequency of 50 kHz, and it is found that, when the switching frequency increases, the efficiency decreases. This is mainly due to the increase in switching losses. In practice, other factors may also affect the performance of efficiency; typical factors include layout construction, the noise level of sensing devices, and driving and control techniques used.

## 5. Conclusions

This paper has presented the systematic design procedure of a GaN-based and digitally controlled high-performance PV emulator that can be widely used in a variety of PV converter studies. Detailed characteristics of PV cells and PV modules have been explained and modeled. The issues regarding controller design and various emulation conditions in terms of normal irradiance and partial shading phenomena in practical solar PV generation applications have been investigated. Based on comprehensive simulation studies and experimental implementation, the proposed GaN-based PV emulator with 50 kHz switching speed exhibits a highest efficiency of 99.05% and excellent dynamic response and accurate current/voltage tracking capabilities. Through this design case, it has been demonstrated that wide-bandgap (WBG) switching devices such as the gallium nitride-based high electron mobility transistors (HEMTs) used in this study offer huge potential for outperforming conventional silicon devices, especially in terms of switching speed and conduction losses. Typical results have been presented to verify the feasibility and effectiveness of the proposed PV emulator.

## Data Availability

No new data were created or analyzed in this study. Data sharing is not applicable to this article.
